# Challenges and Insights: Psychosocial Stressors, Physiology, and Health Disparities

**DOI:** 10.1093/geroni/igaa057.1899

**Published:** 2020-12-16

**Authors:** Julie Ober Allen, Briana Mezuk

## Abstract

The persistence of health disparities demonstrates the need for more comprehensive research to better understand key methodological approaches and intervention leverage points among the complex relationships between psychosocial, environmental, and biological factors influencing patterns of health among older adults. Growing empirical evidence implicates differential exposure to chronic stressors that are rooted in the social environment and related social inequities in premature aging and development of chronic diseases among socially marginalized groups. This, in turn, leads to health disparities within the older adult population. Mechanisms linking stressors and health, however, remain poorly understood. This symposium assembles research examining challenges associated with measuring exposure to stressors, relationships between stressors and health outcomes, and the insight provided by stress-based frameworks for understanding mechanisms of health disparities. Allen and colleagues use comprehensive measures of exposure to stressors to identify stressor characteristics most closely associated with diurnal cortisol dysregulation and Black-White disparities, which are risk factors for adverse cardiometabolic health outcomes. Kalesnikava and colleagues will introduce methods for exploring relationships between self-reported stress and another cortisol stress biomarker—cortisol reactivity to an acute stress event. Byrd and colleagues round out the symposium with a presentation on the directionality of relationships between perceived stress and depressive symptoms contributing to health disparities among Blacks. Discussant Jackson will explore implications of these studies for more nuanced research related to mechanisms of health disparities and for more targeted approaches to the prevention of health disparities among older adults.

